# Psychometric evaluation of the Chinese body shape questionnaire short form for assessing eating disorder symptoms among university students

**DOI:** 10.3389/fpubh.2025.1571144

**Published:** 2025-05-30

**Authors:** Zeng Gao, Jing Zhao, Xi Liu, Haitao Wang, Miaoling Zhang, Han Yuan

**Affiliations:** ^1^Department of Postdoctoral, School of Marxism, Xiangtan University, Xiangtan, China; ^2^Physical Education Department, Xiangtan University, Xiangtan, China; ^3^College of Educational Science, Guangdong Preschool Normal College in Maoming, Maoming, China; ^4^Office of Social Science and Humanities, Xiangtan University, Xiangtan, China; ^5^Department of Physical Education, Kyungpook National University, Daegu, Republic of Korea; ^6^School of Physical Education, Fuyang Normal University, Fuyang, China

**Keywords:** body shape questionnaire, eating disorder symptoms, body shape concerns, psychometric evaluation, reliability and validity, university students

## Abstract

**Background:**

Body shape concerns have emerged in eating disorders as a public health issue among adolescents worldwide. The psychometric properties of the Body Shape Questionnaire (BSQ) remain underexplored in Chinese university students with eating disorder symptoms. This study aims to evaluate the reliability and validity of the Chinese version of the BSQ in the context of eating disorder symptoms among Chinese university students.

**Methods:**

A stratified random sample of 858 Chinese university students (age, mean ± SD = 19.91 ± 1.18) participated in the study. The surveys comprised the BSQ and the EDE-QS to assess body shape concerns with eating disorder symptoms. Eating disorder symptoms were defined as scores equal to or greater than 15 on the EDE-QS.

**Results:**

The Chinese version of the BSQ demonstrated strong internal consistency (Cronbach’s α = 0.92) and robust construct validity. Confirmatory factor analysis supported the original single-factor structure with satisfactory fit indices (Average Variance Extracted = 0.58, Composite Reliability = 0.92, Kaiser-Meyer-Olkin = 0.92, Normed Fit Index = 0.92, Goodness of Fit Index = 0.91, Comparative Fit Index = 0.93, Root Mean Square Error of Approximation = 0.09, Standardized Root Mean Square Residual = 0.03, Tucker-Lewis Index = 0.96). The BSQ showed significant correlations with the EDE-QS (*p* < 0.01).

**Conclusion:**

The Chinese version of the BSQ demonstrates strong psychometric properties among university students with eating disorder symptoms, supporting its use as a reliable and valid assessment tool in the Chinese population.

## Introduction

Body shape concerns (BSC) have emerged as a significant public health issue among adolescents worldwide, particularly in relation to eating disorders (1–3). These concerns substantially impact both physical and mental health, manifesting as anxiety, depression, low self-esteem, and maladaptive eating behaviors including restrictive dieting, excessive exercise, and binge-eating disorder episodes (4–8, 46).

BSC has been consistently identified as both a risk factor for and a maintaining factor of eating disorders, creating a complex bidirectional relationship (9). This aligns with Fairburn’s transdiagnostic model of eating disorders, which identifies BSC as a central maintaining mechanism across various eating disorder presentations (10). Prospective studies demonstrate that heightened BSC significantly associates with an increased risk of developing eating disorders (5, 11–14). Conversely, the psychological burden of eating disorder symptoms further correlates with exacerbated BSC, reinforcing negative BSC and complicating recovery (15–17). Recent epidemiological studies in China reveal that approximately 70.9% of individuals with eating disorders report significant BSC, with 61.9% reporting mild-to-moderate concerns and 9% reporting severe concerns (18).

Multiple factors are associated with the prevalence and severity of BSC, including socio-cultural and environmental elements such as media portrayals of idealized body types, peer pressure, social media influences, family dynamics, and individual psychological factors (19–21). This relationship is particularly pronounced during university years, representing a critical risk period for the development of both restrictive and binge-eating disorder behaviors (5, 12, 22). Research has also indicated that individuals with a higher body mass index (BMI) show increased vulnerability to eating disorder behaviors (23, 24).

BSC are particularly prevalent among individuals with obesity, with manifestations strongly influenced by societal pressures from mass media, family, and peers (4, 12, 25). Social pressures frequently impose unrealistic and largely unattainable body ideals (26, 27), resulting in increased stress and attempts to conform to socially prescribed standards (28, 29). Gender differences in BSC presentations are well documented, with higher prevalence and severity among girls and women (30, 31), while boys and men predominantly exhibit appearance-related behaviors aligned with muscular or athletic ideals (2, 32, 33).

Early identification and intervention are crucial, particularly focusing on improving BSC resilience, fostering self-compassion, and challenging media-driven beauty ideals to prevent the long-term effects of BSC (12, 24). Given the cultural specificity of BSC, it is essential to use culturally and contextually appropriate assessment tools. Despite the growing prevalence of eating disorders in China, there remains a significant gap in validated, culturally appropriate measures for assessing BSC in Chinese populations. This gap is particularly concerning given that most research and measure validation has occurred predominantly in Western contexts.

The BSQ, developed by Cooper et al. (34), is a well-established measure that specifically addresses concerns about body shape and the subjective experience of BSC. The BSQ has been validated across various clinical populations, including not only those with anorexia nervosa and bulimia nervosa but also individuals with binge-eating disorder (35, 36, 52). While the BSQ has been translated and validated in several non-Western countries, including Turkey (37) and Saudi Arabia (38), its psychometric properties have been inadequately studied in East Asian contexts, particularly in China. This represents a significant limitation in the available assessment tools for Chinese populations, especially considering the distinct cultural differences in BSC perception, beauty standards, and eating behaviors between Western and East Asian societies (49). The lack of properly validated Mandarin version instruments specifically tailored to Chinese cultural contexts creates substantial challenges for both clinical practice and research in eating disorders within China.

Although the BSQ was initially translated into Chinese and preliminarily validated by Liao et al. (39), comprehensive psychometric evaluation specifically targeting Chinese university students with eating disorder symptoms (defined as scores ≥ 15 on standardized eating disorder measures) remains notably absent. This represents a significant limitation given the cultural differences in BSC perception and eating behaviors between Western and East Asian populations. Therefore, this study aims to: (1) Evaluate the reliability and validity of the Chinese Mandarin version of the BSQ; (2) Assess its effectiveness in identifying eating disorder symptoms among Chinese university students; (3) Examine the relationship between BSC and eating disorder symptoms in this population.

## Methods

### Participants

Responses were collected from a total of 2,114 Chinese (Mandarin-speaking) university student participants. Seventy-nine questionnaires were deemed invalid due to age accuracy issues (*n* = 9), height accuracy issues (*n* = 2), age below 18 years (*n* = 24), age above 24 years (*n* = 38), or response quality concerns (100% similar responses, *n* = 6). This ensured the validity of the remaining 2,035 questionnaires from participants aged 18–24 years.

Initial screening for eating disorder symptoms was conducted using the EDE-QS, a shortened version of the widely used EDE-Q. Following established guidelines (40), participants with scores ≥15 (out of a possible 36) were classified as having significant eating disorder symptoms. Based on this criterion, 858 participants met the threshold for further analysis.

Our stratified random sampling approach was designed to ensure representative coverage across key demographic variables. The primary stratification variables included gender, academic year (freshman through senior), and field of study (categorized into sciences, humanities, engineering, arts, and medicine). After establishing these strata, we employed proportional allocation to determine sample sizes within each stratum based on their representation in the overall university population. The sampling frame encompassed five universities in central China, with Xiangtan University serving as the primary site (62% of participants) and four additional institutions contributing the remaining sample. A computerized random number generator was used to select potential participants from each stratum using student ID registries provided by university administrations. The initial response rate was 76.3%, with non-respondents being replaced by additional randomly selected students from the same stratum to maintain proportional representation.

All participants voluntarily completed the questionnaires online using the Sojump platform (known as Wenjuanxing). Participants were not compensated for their participation. The study was conducted in accordance with ethical principles consistent with the Declaration of Helsinki. Before participation, all participants provided written informed consent after being thoroughly informed about the study’s purpose, procedures, potential risks, and voluntary nature.

### Measures

#### The body shape questionnaire 8-item short form

The Chinese version of the body shape questionnaire 8-item short form (BSQ-8C) was from Evans et al. (41), who developed the short version of the BSQ. The Chinese version was previously translated and validated (39) and maintains the original unidimensional factor structure, focusing on BSC. Each item is rated on a 6-point Likert scale, ranging from 1 (never) to 6 (always). The total score ranges from 8 to 48, with higher scores indicating greater BSC. Based on the total score, the BSQ-8C categorizes BSC into four levels: no concern with shape (score 8–18); mild concern with shape (score 19–25); moderate concern with shape (score 26–33); and marked concern with shape (score 34–48).

#### The eating disorder examination questionnaire short

The eating disorder examination questionnaire short (EDE-QS) is a 12-item short version of the Eating Disorder Examination Questionnaire, designed to assess key eating disorder behaviors and attitudes. We used the Chinese version validated (42). The EDE-QS measures disordered eating attitudes and behaviors in the past week. The items were rated from zero (0 days/Not at all) to three (6–7 days/Markedly), and a total score is derived by summing and averaging the items, with higher total scores representing more severe eating disorder symptoms. A cut-off score of ≥15 was used as the threshold for identifying significant eating disorder symptoms, following validation studies (40) showing good discriminatory power for this threshold.

### Statistical analysis

Sample size adequacy was determined based on recommendations for confirmatory factor analysis (CFA) (47), with a minimum subject-to-item ratio of 10:1 (43), which our sample (*n* = 858) exceeded for the 8-item BSQ-8C.

Internal consistency reliability was evaluated via Cronbach’s alpha (α ≥ 0.70 considered acceptable, α ≥ 0.80 good, and α ≥ 0.90 excellent), complemented by corrected item-total correlation (CITC) analysis. Additional reliability indices, including Guttman’s split-half coefficient, McDonald’s omega, and theta coefficient, were calculated to provide a comprehensive assessment of scale reliability (48).

The hypothesized unidimensional factor structure was validated through CFA using maximum likelihood estimation in AMOS 24.0. Multiple fit indices were selected to provide comprehensive model evaluation: Comparative Fit Index (CFI > 0.90), Normed Fit Index (NFI > 0.90), Goodness of Fit Index (GFI > 0.90), and Root Mean Square Error of Approximation (RMSEA < 0.10).

Construct validity was verified through convergent validity (Average Variance Extracted [AVE] *≥* 0.50, Composite Reliability [CR] > 0.70) and discriminant validity (comparing high-score and low-score groups using independent samples t-tests). Criterion validity was established by correlating the BSQ-8C with the EDE-QS as an external criterion measure. The significance level was set at *p* < 0.05 for all analyses. All analyses were conducted using SPSS version 26.0 and AMOS version 24.0, with missing data addressed through listwise deletion as it was minimal (< 1% of total data points).

## Results

### Characteristics of the sample

The cross-sectional study enrolled 858 undergraduate students from Xiangtan University and other institutions in China. The cohort comprised young adults with a mean age of 19.91 years (SD 1.18), ranging from 18 to 24 years. BMI distribution revealed that 467 participants (54.43%) maintained normal weight, while 85 students (9.91%) fell into the overweight category and 186 (21.68%) met the criteria for obesity. Notably, 120 participants (12.99%) were underweight. Regarding body shape perceptions, psychological assessments identified 181 students (21.10%) with mild shape concerns, 367 (42.77%) with moderate concerns, and 264 (30.77%) exhibiting marked concerns. The demographics and characteristics show normal distribution expectations through the skewness and kurtosis values. The gender, father’s educational status, mother’s educational status, family income status, and parents’ marital status as a categorical variable did not show normal distribution expectations. Age and BMI as continuous variables. According to the Centre for Health Protection (44), BMI thresholds vary by ethnicity. The Comprehensive demographic characteristics are systematically presented in Table 1.

**Table 1:** Demographics and characteristics (*n* = 858).

Variable	Category	*n*	%	Skewness	Kurtosis
Gender	Male	293	34.15	−1.56	−0.67
	Female	565	65.85		
Age (years)	From (minimum to maximum)	18–24		0.72	0.65
	Median	20			
	Mean ± SD	19.91 ± 1.18			
BMI	Underweight (<18.5 kg/m^2^)	120	12.99	1.88	1.63
	Normal Weight (18.5–22.9 kg/m^2^)	467	54.43		
	Overweight (23.0–24.9 kg/m^2^)	85	9.91		
	Obese (≥25.0 kg/m^2^)	186	21.68		
Father’s educational status	Elementary school	144	16.78	−0.73	0.19
	Secondary school	305	35.55		
	High school	230	26.81		
	Bachelor	175	20.40		
	Master	1	0.12		
	Doctor	3	0.35		
Mother’s educational status	Elementary school	307	35.78	0.11	0.76
	Secondary school	306	35.66		
	High school	167	19.46		
	Bachelor	73	8.51		
	Master	2	0.23		
	Doctor	3	0.35		
Family income status	Low (below 16,443 RMB/Year)	391	45.57	−0.72	0.53
	Average (between 16,443–41,172 RMB/Year)	375	43.71		
	Good (above 41,172 RMB/Year)	92	10.72		
Parents’ marital status	Married	796	92.77	8.98	3.31
	Divorced/widow	62	7.23		
BSQ	NoCS	46	5.36	0.11	0.19
	MiCS	181	21.10		
	MoCS	367	42.77		
	MaCS	264	30.77		
EDE-QS	Mean ± SD	1.73 ± 0.74		2.05	1.35

BMI, Body Mass Index; BSQ, Body Shape Questionnaire; EDE-QS, Eating Disorder Examination Questionnaire Short; NoCS, no concern with shape; MiCS, mild concern with shape; MoCS, moderate concern with shape; MaCS, marked concern with shape.

Correlational analyses demonstrated strong positive relationships between each BSQ item and its total score, with Pearson correlation coefficients ranging from 0.74 to 0.83 (*p* < 0.01). Conversely, statistically significant negative correlations were observed between BSQ items and the EDE-QS total score, ranging from −0.69 to −0.78 (*p* < 0.01). According to the results, indicate robust item-total consistency for the BSQ and divergent validity through inverse associations with the EDE-QS (Table 2).

**Table 2:** Pearson’s correlations (*n* = 858).

Item	Total-score of the BSQ	Total-score of the EDE-QS	CITC	Cronbach alpha if item deleted
BS1	0.81^**^	−0.78^**^	0.74	0.90
BS2	0.83^**^	−0.77^**^	0.77	0.90
BS3	0.80^**^	−0.73^**^	0.73	0.91
BS4	0.74^**^	−0.69^**^	0.65	0.91
BS5	0.82^**^	−0.75^**^	0.75	0.90
BS6	0.78^**^	−0.71^**^	0.71	0.91
BS7	0.79^**^	−0.71^**^	0.72	0.91
BS8	0.80^**^	−0.73^**^	0.73	0.91

^⁎⁎^*p* < 0.01.

At the same time, the BSQ exhibited excellent internal consistency, with a Cronbach’s α of 0.92 (reported in-text as requested). Additional reliability metrics further supported its psychometric robustness: Guttman’s split-half coefficient (0.88), McDonald’s omega (0.93), and theta coefficient (0.92). Item-level analyses revealed strong CITC ranging from 0.65 to 0.77, alongside descriptive statistics (means, standard deviations, skewness, kurtosis, and response frequencies) for each item.

### Item analysis

The item analysis (discriminability) analysis of the BSQ items revealed statistically significant distinctions between high-score and low-score across all eight items (from BS1 to BS8), with critical ratios ranging from 24.908 to 36.827 (*p* < 0.01). Following standard psychometric procedures, the total scores of the eight items were calculated, and participants were divided into high and low scoring groups based on the 25th and 75th percentiles. As presented in Table 3, the high-score group consistently demonstrated elevated mean values (*M* = 4.97–5.51, SD = 0.63–1.15) compared to their low-score counterparts (*M* = 2.60–2.81, SD = 0.77–0.93). Independent sample *t*-tests were performed to compare each item’s mean scores for the high and low groups. The substantial critical ratio values, particularly the highest *t* value of 36.827 for BS1 (high: 5.51 ± 0.63 and low: 2.78 ± 0.93) and the lowest *t* value of 24.908 for BS4 (high: 4.97 ± 1.15 and low: 2.58 ± 0.77). The results indicated statistically significant differences between the high and low scoring groups for all items (*p* < 0.01). Specifically, the *t*-test values demonstrated that each item effectively distinguished between participants with higher and lower overall scores on the measured construct and consistent patterns of significant and strong discriminative validity.

**Table 3:** BSQ item analysis (discriminability) (*n* = 858).

Items	Group (Mean ± S.D.)		*t* (critical value)	*p*
	Low (*n* = 227)	High (*n* = 239)		
BS1	2.78 ± 0.93	5.51 ± 0.63	36.827	0.01^**^
BS2	2.80 ± 0.86	5.37 ± 0.71	35.357	0.01^**^
BS3	2.60 ± 0.77	5.11 ± 0.94	31.510	0.01^**^
BS4	2.58 ± 0.90	4.97 ± 1.15	24.908	0.01^**^
BS5	2.58 ± 0.77	5.10 ± 0.92	32.139	0.01^**^
BS6	2.65 ± 0.83	4.96 ± 1.05	26.350	0.01^**^
BS7	2.67 ± 0.84	4.98 ± 1.07	26.063	0.01^**^
BS8	2.81 ± 0.88	5.11 ± 0.91	27.739	0.01^**^

BS, Body Shape; M ± SD is Mean ± Standard Deviation; ^⁎⁎^*p* < 0.01.

### Construct validity

The questionnaire demonstrated strong convergent validity, evidenced by an AVE value of 0.58 (AVE > 0.50). Internal consistency was exceptional, as reflected in a CR coefficient 0.92. All items exhibited statistically substantial factor loadings ranging from 0.73 to 0.83 (each value exceeding 0.70). Furthermore, commonality estimates ranged from 0.53 to 0.69, all surpassing the minimum acceptable threshold of 0.50. The psychometric results collectively confirm the BSQ’s structural soundness.

The Kaiser-Meyer-Olkin (KMO) measure of sampling adequacy yielded an exemplary value of 0.92, while Bartlett’s Test of Sphericity achieved statistical significance (*χ*^2^ = 4163.24, *p* < 0.01), confirming the suitability of the data for factor analysis. Its CFA further validated the model’s structural adequacy through multiple fit indices, of which the NFI = 0.92, GFI = 0.91, RMSEA = 0.09, SRMR = 0.03, TLI = 0.96, and CFI = 0.93 the values exceeded 0.90. The results showed significant inter-item correlations and satisfactory fit indices.

### Criterion validity

Table 4 showed that the BSQ items had statistically significant correlations (*p* < 0.01) with EDE-QS-TS (*r* = −0.27 to −0.52), revealing an inverse relationship between BSC and eating disorder symptomatology. The inter-item correlation analysis reported that the BSQ total scores and EDE-QS total scores had an association (*r* = −0.88, *p* < 0.01). However, BSQ items showed significant negative correlations (*r* = −0.27 to −0.52) with EDE-QS-TS.

**Table 4:** Inter-item correlations and correlations with total scores (*n =* 858).

	Mean (SD)	BS1	BS2	BS3	BS4	BS5	BS6	BS7	BS8	BSQ-TS	EDE-QS-TS
BS1	5.95 (0.22)	1									
BS2	5.87 (0.34)	0.59^**^	1								
BS3	5.68 (0.71)	0.22^*^	0.37^**^	1							
BS4	5.45 (1.12)	0.01	0.10	−0.01	1						
BS5	5.76 (0.64)	0.20^*^	0.18	0.14	0.07	1					
BS6	5.64 (0.89)	0.01	0.11	0.02	−0.05	−0.05	1				
BS7	5.74 (0.61)	0.13	0.18	0.04	−0.05	0.33^**^	0.25^*^	1			
BS8	5.75 (0.54)	−0.02	0.26^**^	0.01	0.02	0.06	0.21^*^	0.38^**^	1		
BSQ-TS	45.84 (2.39)	0.33^**^	0.55^**^	0.42^**^	0.47^**^	0.46^**^	0.47^**^	0.55^**^	0.46^**^	1	
EDE-QS-TS	15.36 (0.48)	−0.31^**^	−0.52^**^	−0.49^**^	−0.41^**^	−0.37^**^	−0.49^**^	−0.36^**^	−0.27^**^	−0.88^**^	1

BSQ - TS, Body Shape Questionnaire - Total Scores; EDE - QS - TS, Eating Disorder Examination Questionnaire Short - Total Scores; ^⁎⁎^*p* < 0.01; *p < 0.05.

## Discussion

This investigation evaluated the psychometric properties of the Chinese version of the BSQ in relation to eating disorder symptoms among university students. Our analyses revealed several key findings that contribute to understanding the application of BSQ in the Chinese cultural context. The scale demonstrated strong psychometric properties, including good discriminative validity, internal consistency, convergent validity, and satisfactory fit indices. Most notably, we found significant negative correlations between BSQ items and eating disorder symptoms as measured by the EDE-QS, with a strong inverse relationship between total scores. This pattern of negative correlations differs markedly from the typically positive relationships reported in Western samples, suggesting that BSC and eating disorder symptoms may have unique cultural expressions in Chinese university students.

Previous research has established the importance of assessing BSCs in eating disorder evaluations, demonstrating both their significant relationship with clinical symptoms and the multidimensional nature of BSC assessment (4, 6–8, 34). These robust inter-item correlations mirror findings from recent cross-cultural validations demonstrating strong item-total coherence in BSC measures (5, 8) yet contrast with reports of differential item functioning in certain non-Western populations (4). The associations across all BSQ items showed affected a critical psychometric feature in Chinese socio-cultural and environmental factors (19–22). It further reflected constructs of BSC with sociocultural boundaries (3).

Our findings suggest culturally mediated variations in body evaluation schemas and potentially different social desirability biases in symptom reporting among Chinese participants (20, 21). More specifically, the response patterns observed in our Chinese sample suggest that cultural factors may influence how participants interpret and respond to questions about BSC and eating behaviors. Methodologically, all items’ performance reinforces the BSQ’s structural integrity in clinical university students, addressing prior concerns about measurement drift in youth-focused adaptations (9, 24). This finding is significant because it demonstrates that the BSQ maintains its structural validity even when applied to Chinese university students with eating disorder symptoms, supporting its cross-cultural utility. These results support the BSQ’s clinical utility in Chinese settings while highlighting the necessity of developing context-specific norms, as recommended in recent global mental health frameworks (17).

The BSQ’s reliability metrics correspond to thresholds recommended for instruments (2) and align with multinational validations of BSC measures (1, 3). As part of our discriminant validity testing, we intentionally divided participants into high and low score groups (top 27% and bottom 27% of BSQ-8C scores) following established psychometric validation procedures. The significant differences found between these groups were expected and confirm the instrument’s ability to effectively discriminate between varying levels of body shape concerns. The uniform discriminative power of all BSQ items contrasts with findings from cross-cultural adaptations reporting differential item functioning in non-Western populations (4, 18), proving the cultural calibration of the Chinese version. Notably, it reinforces the BSQ’s sensitivity to detect nuanced variations in BSC (6, 7). In addition, the results further support the BSQ’s structural invariance across developmental stages (9, 24). The inter-item coherence corroborates theoretical models positing BSC as a unified construct in eating pathology (21).

The questionnaire reliability indices exceed benchmarks (2), challenging assumptions about measurement attenuation in non-Western populations (4, 18). While the high CR underscores minimal measurement error, given documented reliability declines in translated eating disorder inventories (9, 21, 22, 24). However, it proves cultural calibration of item interpretation despite China’s distinct beauty ideals (5, 8).

The inverse correlations observed between BSQ items and eating disorder symptomatology present a counterintuitive psychometric pattern that challenges conventional Western models of BSC (32, 45). While this aligns with neurocognitive frameworks positing BSC disturbance as a core transdiagnostic feature of eating pathology (31, 33, 51), the negative directionality diverges markedly from established positive BSQ and EDE-QS relationships reported in Western cohorts (1, 3).

This cross-cultural discrepancy may reflect fundamental differences in eating disorder symptom expression shaped by collectivist values in Chinese adolescents, where overt BSC might paradoxically correlate with restrained eating behaviors, a phenomenon documented in Southeast Asian populations (23, 29). It identified subclinical eating disorder presentations (9, 16). At the same time, the strength of their associations exceeds typical BSC, and eating disorder correlations (25, 27). The cultural context of BSC warrants careful interpretation of these findings, particularly regarding the significant negative correlations between BSQ items and EDE-QS total scores. This unexpected inverse relationship challenges conventional Western models of BSC and suggests that as BSC increase in Chinese university students, reported eating disorder symptoms may paradoxically decrease. While the fundamental relationship between BSC and eating pathology appears across cultures, these findings reveal culture-specific expression patterns. The negative directionality may reflect unique manifestations of disordered eating in Chinese contexts, where heightened body awareness might lead to behavioral responses different from those observed in Western populations. Social desirability bias in Chinese contexts may also contribute, as students with pronounced BSC might underreport eating disorder symptoms due to stigma. Chinese cultural values regarding body shape have evolved with increasing Western media influence, yet traditional perspectives continue to influence body shape perceptions in ways that affect measurement relationships (50). These results suggest that assessment approaches developed in Western contexts require significant cultural adaptation when applied to Chinese populations. Nevertheless, the culturally adapted BSQ effectively captured these BSC, demonstrating that properly adapted assessment tools can successfully measure BSC constructs across different cultural settings.

The study has several limitations. Our sample’s focus on university students limits generalizability to other populations. A significant limitation is that we did not include other validated body image measures alongside the BSQ-8C, which limits our ability to confirm the instrument measures body shape concerns as intended. Additionally, our study lacks clinical diagnoses for eating disorders, relying solely on the EDE-QS screening tool. Our psychometric evaluation was limited to basic properties and did not assess sensitivity to change, test–retest reliability, or establish population norms. The gender distribution (65.85% female) reflects an imbalance that likely led to insufficient understanding of male-specific BSC. Our cross-sectional design prevents causal inferences, while reliance on self-report measures introduces response biases, particularly social desirability in collectivist cultures. Future research should address these limitations with longitudinal, multi-site designs, clinical diagnoses, and culturally adapted diagnostic frameworks, while incorporating mental health assessments and gender-specific analyses.

## Conclusion

The study evaluated the Chinese version of the body shape questionnaire with eating disorder symptoms among university students. The findings demonstrated strong psychometric properties, including high internal consistency, statistically significant distinctions between high-score and low-score groups, good convergence validity, and high CR coefficient. These results highlight the accuracy and significant relationship between body shape concerns and eating disorder symptoms, supporting the use of this questionnaire as a reliable and valid assessment tool in the Chinese population.

Future research can build upon this foundation by exploring the cultural dimensions of body image concerns in China, particularly the intriguing negative correlation observed between BSQ and eating disorder symptoms that differs from Western findings. Cross-cultural studies comparing measurement properties and incorporating China-specific body image concepts would enhance our understanding of these cultural differences. Including more diverse samples across different regions of China and examining gender-specific manifestations would provide more comprehensive insights into body shape concerns in Chinese populations.

## Data Availability

The original contributions presented in the study are included in the article/supplementary material, further inquiries can be directed to the corresponding author.
